# Impacts of an abbreviated personal agency training with refugee women and their male partners on economic empowerment, gender-based violence, and mental health: a randomized controlled trial in Rwanda

**DOI:** 10.1186/s12889-024-18780-8

**Published:** 2024-05-14

**Authors:** Naira Kalra, Lameck Habumugisha, Anita Shankar

**Affiliations:** 1 Africa Region Gender Innovation Lab, Office of the Chief Economist, The World Bank Group, Washington D.C., USA; 2 Plan International Rwanda, Kigali, Rwanda; 3 Department of International Health, Johns Hopkins University, Bloomberg School of Public Health, 615 N. Wolfe Street, Baltimore, MD 21205 USA

**Keywords:** Gender-based violence, Intimate-partner violence, Randomized controlled trial, Economic empowerment, Evaluation, Personal agency, Mental health, Clean cookstoves

## Abstract

**Introduction:**

We assessed the impact of a personal agency-based training for refugee women and their male partners on their economic and social empowerment, rates of intimate partner violence (IPV), and non-partner violence (NPV).

**Methods:**

We conducted an individually randomized controlled trial with 1061 partnered women (aged 18–45) living in a refugee camp in Rwanda. Women received two days of training, and their partners received one day of training. The follow-up survey where all relevant outcomes were assessed was carried out at 6–9 months post-intervention.

**Results:**

At follow up, women in the intervention arm were more likely to report partaking in income generating activities (aIRR 1.27 (1.04–1.54), *p* < 0.05) and skill learning (aIRR 1.59 (1.39–1.82), *p* < 0.001) and reported a reduction in experience of physical or sexual NPV in the past six months (aIRR 0.65 (0.39–1.07), *p* < 0.09). While improved, no statistically significant impacts were seen on physical or sexual IPV (aIRR 0.80 (0.58–1.09), *p* = 0.16), food insecurity (β 0.98 (0.93 to 1.03), *p* = 0.396), or clean cookstove uptake (aIRR 0.95 (0.88 to 1.01), *p* = 0.113) in the past six months. We found statistically significant reduction in physical and sexual IPV amongst those experiencing IPV at baseline (aIRR 0.72 (0.50 to 1.02), *p* < 0.07). Small improvements in self-efficacy scores and our indicator of adapting to stress were seen in the intervention arm. Some challenges were also seen, such as higher prevalence of probable depression and/or anxiety (aIRR 1.79 (1.00-3.22), *p* = 0.05) and PTSD (aIRR 2.07 (1.10–3.91), *p* < 0.05) in the intervention arm compared to the control arm.

**Conclusion:**

Our findings echo previous research showing personal agency training can support economic well-being of women. We also find potentially promising impacts on gender-based violence. However, there is some evidence that integration of evidence-based mental health support is important when enhancing agency amongst conflict-affected populations.

**Trial registration number:**

The trial was registered with ClinicalTrials.gov, Identifier: NCT04081441 on 09/09/2019.

## Key message

**What is known?**

• There are limited impacts of economic interventions in humanitarian settings on gender-based violence.

• Outside of humanitarian settings, agency-based training interventions, both with/or without male partner engagement, have been shown to improve economic impacts, however IPV impacts are not known.

• Integrated multi-component interventions that economically empower women and engage male partners hold promise in conflict-affected populations.

**What are the new findings?**

• An abbreviated two-day personal agency training for women and one-day training for their male partners led to significant increases in uptake of income generating activities and skill building for women.

• Promising trends suggest reduction in non-partner violence for the full study sample and a reduction in intimate partner violence for women who reported IPV at baseline.

• Increased rates of probable anxiety and/or depression and post-traumatic stress disorder (PTSD) were identified in the intervention group.

**What do the new findings imply?**

• Promising impacts on livelihoods and experience of violence are possible despite the abbreviated nature of this training.

• Despite improvements in livelihoods and reduction in experience of violence, more concerted efforts are needed to prevent the increased risk of anxiety, depression, and PTSD found in conflict-affected populations.

## Introduction

Economic insecurity, mental distress, and violence-related vulnerabilities are heightened amongst refugee women. Rates of violence perpetrated by intimate partners are higher than rates of wartime physical or sexual violence at the hands of non-partners [1]. It is estimated that nearly one in five female refugees has experienced physical and/or sexual violence by an intimate partner [2, 3]. This experience of intimate-partner violence (IPV) is associated with adverse health and well-being outcomes, including injury, sexually transmitted diseases, and worsened mental health amongst women in refugee camps [4–6]. In addition to IPV, women in humanitarian settings also face violence from non-partners. For example, foraging for firewood for cooking needs in refugee camps has been identified as a prevalent risk factor for non-partner violence (NPV) [7]. The multiple challenges faced by women in such settings requires integrated, often multilayered interventions.

Economic distress is known to exacerbate violence and is viewed as a modifiable risk factor in refugee settings [8]. The refugee populations in Rwanda tend to be in a protracted situation i.e. have lived more than 5 years in the host country and despite having a right to work, struggle to integrate into the job market in the host community [9], thus experiencing economic distress. Prior research has focused on refugee women’s economic empowerment, largely through sustainable approaches such as microcredit or savings groups programs, with or without a social norms component, as a key approach to address economic insecurity, reduce women’s risk of experiencing IPV, and improve their mental health [10]. While some of these interventions were successful in improving livelihoods, gender attitudes, mental well-being, and economic well-being, these programs typically found no statistically significant impacts on women’s experience of physical and/or sexual IPV and did not assess impacts on non-partner violence (NPV) [10, 11].

Another approach to addressing GBV shown to successfully reduce rates of IPV outside of humanitarian settings includes adapting programs that used group learning and engaged partners through community gender dialogues [12, 13]. Recent evidence from the ‘safe at home’ trial in the Democratic Republic of Congo (DRC) finds that single-sex discussion groups for couples significantly reduced the risk of IPV for women and harsh discipline for children [14]. However, in another study amongst conflict affected populations in the DRC, similar gender dialogue trainings with men alone have not been found to reduce IPV [11]. Moreover, engaging men led to no promising impacts on women’s economic empowerment and did not address NPV. These interventions are also extremely resource and time intensive and require participants to attend upto 29 weekly sessions over a period of 6–8 months [14].

To find innovative, less resource intensive, and feasible solutions to address the complex problem of poverty and gender-based violence, we turned to qualitative research from Rwandan refugee camps which suggest that an empowerment approach is needed as part of any efforts to address violence as it strengthens women’s voice and agency, something that is lacking in current approaches [15, 16]. The broader evidence outside of humanitarian settings also suggests that economic empowerment, and especially economic empowerment and social empowerment programs when combined can be effective in reducing IPV [17, 18]. The ‘IMAGE intervention’ [19], tested a micro-finance program in South Africa paired with 10 one-hour sessions of participatory trainings on health, gender norms, communication, leadership, and gender-based violence called ‘sisters for life’ and showed a significant reduction in experiences of IPV. Similarly, an economic and social empowerment intervention implemented in 24 sessions over 12 months combined with a cash-transfer component in Afghanistan was successful in reducing IPV amongst those experiencing moderate food insecurity prior to the intervention [20] and in DRC a similar 12-month intervention was successful in reducing IPV amongst those at higher risk for IPV at baseline [21]. These interventions indicate the potential of empowerment interventions but did not unpack whether these effects would exist in the absence of the micro-finance and/or cash transfer component.

Agency-focused empowerment trainings, often referred to as personal agency or personal initiative trainings, have been previously shown to improve women’s personal and economic outcomes in populations not affected by conflict. Their effectiveness within conflict-affected populations and its impacts on GBV, especially IPV, and mental well-being remain understudied. These behavioral interventions, based on principles of psychology and neuroscience, have been shown to enhance the profits and psycho-social measures of agency in female entrepreneurs in both Kenya and Togo [22–24] and more recently, found to increase spousal support for business activities and improve partner relations [25]. A recent evaluation of the *‘*Adolescents: Protagonists of Development’, a personal agency and economic empowerment training paired with technical skills training found positive impacts on both economic well-being and reduced the risk of violence experienced by adolescent girls in Bolivia [22]. While this approach appears promising, with 64 h of training [22] some of these are also resource and time intensive programs that are potentially difficult to scale and sustain in a humanitarian setting. In addition to the need for efficient allocation of scarce rescources, feasibility testing of interventions with refugee population often results in abbreviating programs further indicating that longer programs are not desirable in this setting [26, 27]. Additionally, despite shortening their intervention to just seven sessions, Greene et al., (2021) find that the participation continued to drop with every session and only 33% of refugee women attended all sessions [28].

Unlike approaches that involve shifting norms, some agency approaches can be delivered successfully in a shorter period of time [19, 24]. Keeping in mind that some relatively shorter programmes that focus on agency building were also found to be effective in reducing IPV, we examined the impacts of an abbreviated personal agency training with women and their male partners on GBV and women’s social and economic empowerment. Our approach differs from other programs, both in content and duration. Compared with personal initiative interventions, deployed over several months and focused specifically on goal setting on one’s business, our program focused on using an abbreviated 2-day personal agency intervention with women followed by a 1-day personal agency training with their male partners to enhance multiple aspects of one’s life and targeted a multitude of GBV risk-factors. The intervention was structured to guide individuals through a process of self-reflection, identification of personal aspirations and strategies for action within their socio-cultural and contextual constraints. While this process was individualized, it was conducted within a group framework to leverage collective agency. The objective was to enhance collaboration between women and their male partners, who underwent separate reflective processes and foster more effective pursuit of shared goals upon reunification.

We included a gender-sensitive male engagement component in the refugee setting to counter men’s sense of failure and emasculation that might result from the perception of women’s enhanced economic empowerment that may have led to backlash [29, 30]. This decision was informed by advice from refugee camp leaders and evidence from programs that integrate engaging partners and economically empowering women that may have promise in reducing GBV and improving livelihoods in conflict-affected settings compared to economic empowerment alone [31, 32]. Other studies with similar populations, such as the Nguvu trial with female Congolese refugees in Tanzania, report participants suggesting that their male partners be involved in the intervention and that services be provided for men as well [27]. Based on their study in post-conflict Uganda, Green et al. (2015) suggest a light touch engagement of men in women’s empowerment interventions as they found a one day training for male household members on gender-relations, communication and problem solving was effective in improving the quality of the relationship [32]. Furthermore, we integrated exercises on task sharing and clean cooking adoption to address women’s risk of NPV.

## Methods

### Study setting and trial design

The Kigeme camp, located in Nyamagabe district about 150 km from Kigali, opened in 2012 and is home to 17,681 Congolese refugees in 3,366 households [33]. The camp is structured around two administrative layers, quarters and villages, each having its own elected representatives resulting in eight executive and quarter leaders and 27 village leaders. The camp is administered by MINEMA, which is responsible for the security and protection of the refugees in coordination with UNHCR. The study was carried out in collaboration with Plan International, Rwanda, which was responsible for social protection and GBV response in the camp at the time of planning the study (2018–2019). Multiple stakeholders provide additional services in the camp, including protection, food, WASH, GBV, education, and health [33].

Local staff in the refugee camp and UNHCR staff members in the local offices were apprised and consulted before and during key aspects of study implementation. Plan staff engaged local community leaders and presented both these studies to the community at their monthly meeting before any activities began and throughout the project. In collaboration with other international NGOs and service providers in the camp, a referral network for IPV and mental health support was established. All research activities were approved by the Institutional Review Board at the Johns Hopkins University, Bloomberg School of Public Health (USA) approval number IRB00009381 and the Rwanda National Ethics Committee (RNEC). Further approvals were obtained from the National Center for Science and Technology (NCST), the Ministry of Education (MINEDUC), and MINEMA, Rwanda, for every year the study was active.

We carried out a two-arm, individually randomized controlled trial with partnered women in Kigeme refugee camp in Rwanda to study the impact of an abbreviated personal agency-based intervention. All the women recruited into the study at baseline were randomized using a computer-generated list to either intervention or control arm on a 1:1 ratio (generated in SAS version 9.4; SAS Institute Inc. 2013. SAS® 9.4 Statements: Reference. Cary, NC: SAS Institute Inc.). This study was originally planned as a 2 × 2 factorial design with one RCT designed to examine the impacts of clean cookstove adoption on gender-based violence and another RCT where a smaller sub-set of partnered women were cross randomized to either the personal agency-based intervention or control group. This would have resulted in four groups: clean cookstove adoption + personal agency training, personal agency training alone, clean cookstove adoption and control/waitlist. However, internal changes in policy in the camp and delays in permit renewal led to a shift in the timeline. Clean cookstoves were offered to all residents of the camp by March 2019. This was just after the roll-out of the personal agency-based intervention. Therefore, at the time of the follow-up for this study in August/September 2019, both arms of this study had several months of equal access to adopting clean cooking solutions and for all practical purposes this acts like a two-arm trial.

### Sample size

Sample size calculations used estimates of partner violence obtained by a prior study amongst Congolese refugees in Rwanda, reporting a prevalence rate of 22% for IPV [6] and were calculated to detect a 35% difference with an 80% power and significance level (alpha) of 0.05. Despite a short period of post-intervention follow-up, we anticipated attrition due to rapid movement from the camp and accounted for 20% drop-out, resulting in an estimate of 502 participants needed in each arm of our study.

### Identification and selection of participants

Locally hired recruiters from within the refugee camp went home to home and in line with WHO’s ethical guidelines on measuring IPV, recruited one woman from each household based on eligibility criteria. Participants were informed that they would be participating in a research study and would be randomly selected to be offered a clean cookstove and/or be selected to participate in an upcoming empowerment training program. Eligibility criteria were as follows: participants were female, between 18 and 45 years, currently living in the refugee camp, and living there for the past year, with no intention to relocate in the next year. Only those who reported living with an intimate partner for the last six months or more were included in the agency-based training.

The study was implemented between August 2018 and September 2019, with a baseline conducted between August and September 2018. Households/women were recruited for both studies simultaneously. Separate random allocation (of the full sample) to both interventions (the encouragement to adopt clean cooking solutions intervention and the personal agency training intervention) of all eligible households/women was carried out prior to baseline data collection. All the women recruited to the study completed a baseline survey. We applied our eligibilty criteria to 2000 women. Of these, we removed one duplicate, 847 women reported that they did not currently have and intimate partner and 91 reported that they had not lived with their partner at all in the past six months. This sample of 1061 formed the baseline of the personal agency study and from amongst this sample, those already randomized to the personal-agency intervention after recruitment were invited to the training and the remaining formed the control group. The intervention was deployed between December 2018 and February 2019. Of those selected and offered the training, 9.7% did not attend the training. All women were provided referrals to mental health and GBV support services within the camp at the end of the survey.

**Fig. 1:**
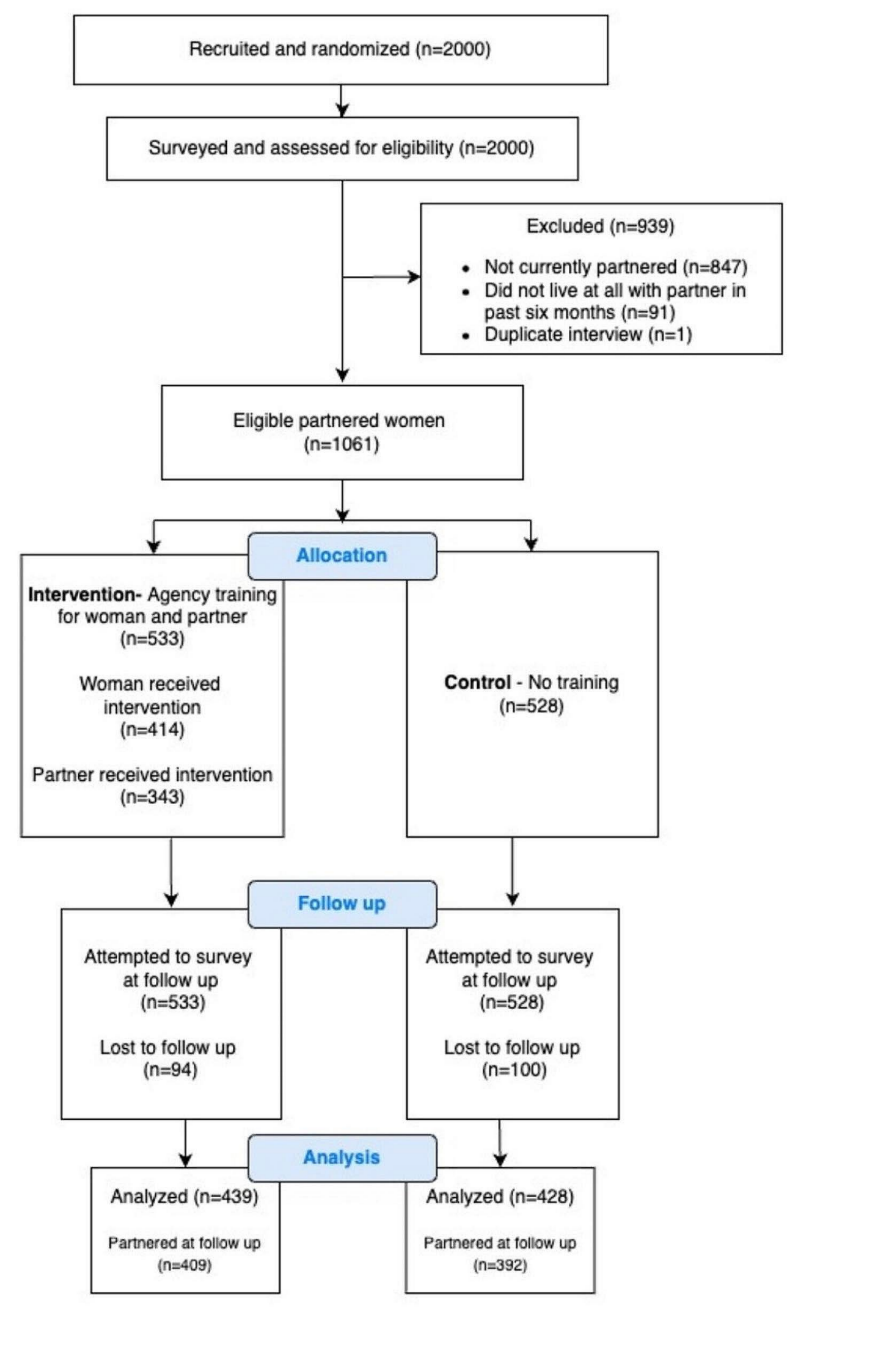
Flow of participants through the study

A follow-up survey was to be carried out with the 1061 women who were eligible for the personal agency study six months after the last group of women received the intervention. However, 18.3% of our sample was lost to follow-up, primarily because the individuals could not be found, with no significant difference (*p* = 0.583) in drop-out between intervention and control groups. At follow-up, 66 women reported no longer being in a relationship and were subsequently not asked IPV questions. Figure 1 illustrates the flow of participants though the study.

### Intervention

The Nimenye Mpinduke, Nigire (NMN) training is an adapted version of the personal agency training developed by the Self-Empowerment and Equity for Change Initiative (SEE Change), specifically designed for the Rwandan context. Its aim is to increase personal awareness of thoughts, beliefs, and past actions and their impacts on future behaviors, effectively enhancing personal agency. The study’s unique feature is the inclusion of male partners in a shortened one-day training, developed in collaboration with the Rwanda Men’s Resource Centre (RWAMREC) and focused on positive masculinity and male engagement approaches. The NMN intervention was adapted from SEE Change’s open-source Empowered Entrepreneur Training Handbook (EET). Adaptation of the original 32 h of personal agency and leadership content was done in a two step process. First, in collaboration with Rwandan colleagues at Plan International and RWAMREC, the team selected key exercises that would be applicable for a humanitarian context (approximately 20 h). We then engaged 14 Congolese female and 12 male refugees in Kigeme camp to serve as trainers, continuing to customize content over three weeks as part of the TOT activities in November 2018. This content was further abbreviated and outlined as two 6-hour sessions for women and one day for men. This included separate discussions with women and men to tailor the content to their specific needs. Joint sessions followed to deepen understanding to reflect the context of the refugees’ experiences in the camp. Trainers then piloted and refined the content before the intervention was deployed. Men and women attended separate workshops as the emphasis was on developing individual resilience and agency while exchanging personal experiences. In a mixed-gender workshop, prevailing power dynamics and societal norms might discourage participants, especially women, from freely sharing vulnerabilities and openly discussing such matters. While women were not asked about IPV or NPV directly, it was always possible that it came up. Therefore, we believed it was best that any disclosure did not happen in front of the partner.

The female participants underwent approximately 12 h of training conducted over two consecutive days, incorporating individual exercises and interactive group discussions drawn from positive psychology techniques such as cognitive behavioral therapy, mindfulness, and meditation. Based on previous pilots done in the region, we learned the content is best delivered in an intensive way (e.g. over one or two days consecutively) to allow individuals to experience their personal journey and reinforce the concepts by reflecting on various areas of their life, led by trainers were sourced from the community who understood the socio-cultural context and the lived experience of the participants. The training began with exercises designed to increase awareness of one’s life journey and hopes and dreams for the future. Participants learned tools to help reframe negative thought patterns and identify clear, doable actions to move forward within different life domains, reinforcing this positive focus in their communications and actions towards others. Male partners underwent a six-hour, shortened version of the NMN training with exercises developed in conjunction with RWAMREC, a local non-governmental organization (NGO) working with men and focused on the promotion of positive masculinity and male engagement approaches. This NGO had previously developed the intervention for two other successful gender dialogue programs in Rwanda [12, 13]. The training began with a competition between two groups of participants to make tea using a traditional firewood stove and the clean cookstove and fuel system, followed by a discussion on gendered task divisions and benefits of clean cooking solutions. The training included exercises to examine one’s life, the ways their thoughts and beliefs influence their behaviors, and ways to reframe negative thought patterns. The workshop concluded with a session on positive communication within the household. One key aim of training male partners was to reduce the risk of NPV during firewood collection by supporting the improved uptake of clean cooking systems. Table 1 summarizes the key components of the intervention.

All trainers were selected from refugees currently residing in the camp, with female trainers trained for five days over the course of two weeks and male trainers trained for three days over the course of one week. The last half-day of training included a joint session with female and male trainers, allowing for the sharing of experiences, ideas, and discussions.

**Table 1:** SEE Change, Nimenye, Mpinduke, Nigire (NMN) content exercises

Female engagement	
Conducted over two days (approximately 12 h of content)	
Session Title	Goals
1. Introduction	Introduce the training, create a community agreement.
2. Think-Feel-Do	Explore the relationships between how we think, how we feel and what we do. Have individuals understand the importance of self-awareness and the ability to modulate thought.
3. Sex and Gender	Explain the distinctions between one’s biological sex and the societal norms related to gender. Have participants explore how the lives and work of women differ from those of men in the community.
4. Myself-My Friend	Give participants the opportunity to think about themselves as their friend and understand that by caring for themselves, they will be better able to care for others and do the things they want in their lives.
5. Tree of Life	Individuals review their life, with the roots of the tree representing their history, the trunk representing today, the fruits representing their accomplishments, and the buds representing their dreams they have for their future. This serves as the starting point for where they want to go in the future.
6. Limiting Beliefs and Reframes	Explore the concept of limiting beliefs – beliefs that we have that make us doubt ourselves or what we are capable of. These are beliefs that we have the power to change. A reframe is a substitute to the limiting belief that acknowledges our positive potential.
7. Core Beliefs	Six core beliefs and potential limiting beliefs that people may have related to these Core Beliefs are explored. (1) Self-responsibility – the belief that we have control over our thoughts and actions. (2) Self-esteem – how you think about yourself and the belief you have of your own worth. (3) Trust in a higher purpose – being willing to have trust in a purpose that is larger than ourselves. (4) Positive attitude –the willingness to focus on the good things in a situation, to find opportunities and constructive solutions in whatever life presents. (5) Continual growth through life – accepting that everything in life is always changing. (6) Owning your power + positive power – recognizing that we are the most powerful person in our own lives and being careful about how we wield power.
8. Personal Behavior Change	A five-step process to identify a small next step towards one’s goals. This includes (1) Awareness: What is my current behavior, and what is the specific challenge I face? (2) Meaning: Why is it important to me to change this specific behavior? (3) Vision: How would I like to act or be? (4) Mind shift: How do I change how I think about this issue to help me move towards my vision. (5) Growth step: What is my next clearly stated next step to move forward to get to my vision?
9. Letting Go	Practice letting go of past hurts so that we can help make space for new thoughts and beliefs and address unhelpful fears we may hold. This process can be the beginning of letting go of those thoughts/memories of past hurts, so that we can start building stronger selves.
10. Body Dialogue	A movement exercise accompanied with a visualization to increase awareness of our bodies and things that impact good health, including food, drink, pollution, and the need to be aware of sources of poor health.
11. Positive Communications	A role play exercise to explore ways of communicating with others and practice effective communication.
12. Closing	Commitment to action
**Male engagement**	
Conducted over 1 day (Approximately six hours of content) and co-facilitated by RWAMREC	
1. Introduction	Introduction to the shortened SEE Change training
2. Cooking Activity	With men split into two groups, we explore different methods of cooking tea (one with a clean cookstove and fuel and one with traditional firewood three stone fire) followed by discussion on the benefits and harms of these approaches for women and the household.
3. Gender Box and Gender Roles	To examine the different roles women and men have in society and how that affects everyday life.
4. Think-Feel-Do Cycle	Same as the content for women (but shorter time)
5. Limiting Beliefs and Reframes	Same as the content for women (but shorter time)
6. Core Beliefs	For men, we explore eight core beliefs, including the six beliefs mentioned above in the women’s training and an additional two gender beliefs: [1] Having more gender equal beliefs and recognizing the value of equality [2]. Positive relationships, recognizing the value of positive relationships and peace in the household for yourself and your family members.
7. Healthy Emotions and Communication	Expressing emotions and dealing with anger: To identify differences in the ways men and women express emotions; to explain consequences of not expressing emotions; and to identify and practice strategies for reacting constructively and non-violently when angry.
8. Letting Go	Same content as for women
9. Positive Communications	Same content as for women with an additional focus on alcohol facilitated abuse and control behaviors
10. Cognitive Reframing – Negative to Positive	Mental tools to move thoughts from negative towards more positive thoughts.
11. Closing	Commitment towards action

### Outcomes

Table 2 summarizes the key outcomes assessed in the study. We registered the protocol at ClinicalTrials.gov (identifier: NCT04081441) in line with the original study plan, which was developed prior to beginning field activity. Some modifications were made prior to baseline data collection and randomization. The Generalized Health Questionnaire (GHQ) was removed from the survey to shorten its length. The Hopkins Symptom Checklist (HSCL-25) [34] and the Harvard Training Questionnaire (HTQ) [35] were retained, as these measures are more specific to domains of mental health problems particular to these settings and that these measures have been validated with this particular DRC population, by Bass et al., while the GHQ has not [36]. The food insecurity experience scale (FIES) was replaced with the household food insecurity access scale (HFIAS) [37], which reports food insecurity at the household level instead of the individual level. We replaced Duckworth et al.’s measure of Grit with the Shift and Persist measure, as the former references ‘projects’ and ‘shifting interests’ and hence did not apply well to the context of refugee camps [38].

**Table 2:** Description of outcome variables

Variable	Variable Construction and Source	Scaling	Hypothesized Direction	Source
Physical and/or sexual IPV in the past six months	Binary variable coded as ‘1’ if a female participant with an intimate partner responded affirmatively to experiencing any act of physical or sexual violence from an intimate partner in the past six months and ‘0’ if no act was experienced. Acts of physical violence included slapping, pushing, twisting her arm or pulling her hair, hitting her, kicking or dragging her, choking or burning on purpose, or using weapons. Acts of sexual violence included forced sex, respondent’s performing sexual acts out of fear, performing of sexual acts found humiliating/degrading by the respondent, forced sex with threats.	Binary	Decrease	Items based on the WHO Violence Against Women Scale used in the WHO multi-country study [39]
Emotional IPV in the past six months	Binary variable coded as ‘1’ if a female participant with an intimate partner responded affirmatively to experiencing any act of emotional violence from her partner in the past six months and ‘0’ if she reported not experiencing such acts. Acts include the following: insulted, humiliated, scared or intimidated, threatened to harm, prohibited from seeing friends or family, being kicked out of her home, refused money for household expenses when it was available, or if the respondent reported she was afraid of her partner.	Binary	Decrease	Items based on the WHO multi-country study and pilot testing of instruments [39]
Reproductive coercion in the past six months	Binary variable coded as ‘1’ if the participant responded ‘yes’ to if a current husband/partner ever refused to use a method or tried to stop her from using a method to avoid getting pregnant in the past six months and ‘0’ if she said ‘no’.	Binary	Decrease	Items based on the WHO multi-country study and pilot testing of instruments [39]
Harassment in the past six months	Binary variable coded as ‘yes’ if a participant responded affirmatively to experiencing any acts of harassment in the past six months. Based on the following acts: catcalls (sounds like kissing sounds, lip smacking, whistles), unwanted attention, undesirable interaction, remarks or come on, crude or offensive jokes and attempts to humiliate, stranger entered home uninvited and made the respondent feel uncomfortable, unwanted touching, stroking or hugging, indecent exposure or ejaculation, or stalking.	Binary	Decrease	Self-reported
Non-partner sexual or physical violence	Binary variable coded as ‘1‘ if a participant responded affirmatively to experiencing any acts of physical or sexual violence in the past six months from someone other than their partner and ‘0’ if no acts were experienced. Acts of NPV include forced sex or sexual activities, unwanted forceful fondling or grabbing, threatened sexual violence or rape, attempted rape, attack with a weapon, kicked, dragged or beaten, slapped, twisted arm, hit, pushed, or shoved.	Binary	Decrease	Items based on the WHO multi-country study and pilot testing of instruments [39]
Clean cookstove uptake	At baseline: self-reported clean cookstoves use along with the customer identification number. At endline: self-reported and verified cookstove customer in August 2019. Binary variable coded as ‘1’ for self-reported uptake and verified user and ‘0’ for verified non-cookstove user and self-reported no-uptake in the past six months.	Binary	Increase	Verified through purchase data and self-reported use at endline
Women’s engagement in income- generating activities	Binary variable assessing women’s self-reported income generating activities. Coded as ‘1’ if participant responded affirmatively that she was engaged in activities to earn money in the past six months at endline and ‘0’ otherwise. At baseline, we derive this from income generation in the past one month.	Binary	Increase	Self-reported
Women’s engagement in skill development	Binary variable coded as ‘1’ if woman self-reported starting to engage in learning a new skill in the past six months and ‘0’ otherwise.	Binary	Increase	Self-reported
Probable depression and anxiety	Anxiety and depression score generated based on responses to the Hopkins Symptom Checklist-25 (HSCL-25). Responses were summed and standardized to obtain a mean score and converted into a binary variable with a standardized cut-off of 2 as suggested by the authors of the measure. Participants responded to the frequency of experiencing symptoms of anxiety (10-items) and depression (15 items) in the past one month.	Binary	Decrease	Hopkins Symptom Checklist-25 (HSCL) [34, 36]
Probable PTSD	Trauma score generated using the Harvard Trauma Questionnaire (HTQ), comprising 16 items assessing trauma-related symptoms experienced in the past one month. Responses were summed and standardized to obtain a mean score and a cut-off of 2 was used to create a binary variable.	Binary	Decrease	Harvard Trauma Questionnaire (HTQ) [35]
Food insecurity	Food insecurity score generated using the nine-item Household food insecurity Index (HFIAS). Scale assessed average household food insecurity in the past one month. Items asked about frequency of respondent’s fear about lack of enough food; inability of household members to eat preferred foods; household members eating limited variety of foods; household members limiting quantity of food; household members reducing frequency of food; having no food; whether any household member went to sleep hungry and whether anyone in the household did not eat day or night. Each item had four responses related to frequency of food insecure behavior ranging from none, rarely, sometimes, and often (scored as 0–3). These were summed to obtain a food insecurity score ranging from 0 to 27. Higher scores indicate greater food insecurity. Sensitivity analyses were carried out with binary outcomes indicating severe food insecurity based on the scoring suggested by Coates et al. (2007) [37].	Continuous	Decrease	Coates et al. [37] Developed by the Food and Nutrition Technical Assistance Project (FANTA) at the US Agency for International Development
Self-efficacy (Chen et al.) index	Self-efficacy was assessed using eight items, which measure individual self-efficacy using a 4-point Likert scale. Items summed to make a self-efficacy score with a range of 1 to 32. Higher scores indicate greater self-efficacy	Continuous	Increase	Chen et al. New General Self-Efficacy Scale (2001) [40]
Shift and Persist Scale	Fourteen-item scale with four items assessing the construct of ‘shifting’ or adapting to stress by measuring how the participant copes with stressful or undesirable situations [41]. These ask about learning from a stressful situation, thinking about good from a stressful situation, thinking about good that can come from a situation that doesn’t turn out how the respondent wants it to and learning from a situation. Four items assess the construct of ‘persisting’ or being able to retain an optimistic outlook in the face of adverse situations [41].	Continuous	Increase	Shift-and-Persist Strategies [41]
Social agency – environmental mastery and positive relations	Social agency was measured using six questions from two Ryff subscales [42]. The first one assessed environmental mastery, i.e. the ability to manage complex situations, feeling in charge of them, and managing day-to-day responsibilities and feelings about demands of daily life. The second three-item sub-scale assessed maintaining positive and trusting relationships with others, loneliness due to few close friends, and whether the respondent identifies as a giving person willing to share time with others. Three negatively worded statements were reverse scored, and all items were summed to create a score on scale with a range of 6 to 24. Higher scores corresponding with greater agreement with statements endorsing mastery or positive relations.	Continuous	Increase	Ryff Psychological Well-being Scale [42]
Income generation	The amount noted in response to asking women who reported working in the past six months: “How much total money (gross income) did you (woman) earn in the last month in cash and in kind, excluding the amount received in cash transfers?”	Continuous	Increase	Self-reported
Physical punishment towards children	Items from the Multiple Indicator Cluster Survey (MICS) child discipline module, including whether or not they used any of physical methods of disciplining their child in the past month such as shaking, spanking, hitting the child. Binary response coded as ‘1’ if respondent reporting using any of these methods and ‘0’ if they said they did not use these.	Binary	Decrease	Multiple Indicator Cluster Survey (MICS) child discipline module as used by Doyle et al. [12]
Women’s report of men’s participation in childcare	Women’s report of how she and her partner distributed childcare in the past six months. Binary variable coded as ‘1’ if woman reported sharing childcare equally with the partner or if it was usually or always carried out by the partner and ‘0’ otherwise.	Binary	Increase	Self-reported

### Data analysis

Chi-squared tests were used to examine differences between intervention and treatment arm at baseline. At follow-up, an intention-to-treat analysis on the sample that was not lost to follow-up was carried out with all women who participated in both baseline and six months endline analysis. Generalized linear models (GLM) compared outcomes between control and intervention arms [43]. For binary outcomes, the econometric specification involved using a Poisson distribution and a log link. For continuous outcomes, a Gaussian specification with a log link was used. Robust standard errors were specified. We carried out both adjusted and unadjusted analysis. In the adjusted analysis, we adjust for woman’s age, education, and baseline value of emotional IPV, as these were imbalanced at baseline and likely to be associated with all outcomes assessed. We also adjusted for the baseline value of the outcome, except for the Shift and Persist score and the engagement in skill learning outcome, which were not assessed at baseline.

We included some key outcomes that had been explored in recent impact evaluations of socio-economic or couple’s interventions, such as impacts of the intervention on those experiencing IPV at baseline [13], impacts of the intervention on physical punishment towards children and sharing of childcare duties [12], and past month income [20]. In addition to making our study comparable with the latest literature, we also believed that IPV amongst those experiencing partner violence at baseline was a more meaningful measure as we expected empowerment to result in breaking the existing cycle of violence. Income was a relevant measure and one that would have changed directly because of women’s economic empowerment. We also believed that physical punishment towards children could change due to potential reduction in IPV, NPV and improvements in mental health [44, 45]. Furthermore, since RWAMREC also developed ‘Bandebereho’ [12], sharing of traditionally female tasks such as child care duties remained a topic of focus for the ‘Gender Box’ activity and the gender role discussion, as well as for the gender core beliefs materials developed by them and hence was a meaningful outcome for this study as well. We used Stata (V.14) for the data analysis [46].

## Results

Table 3 describes the socio-demographic characteristics at baseline and Table 4 provides an overview of baseline values of outcomes for both intervention and control samples. Women in the intervention group were slightly older (33.4 years vs. 32.7 years) (*p* = 0.09) and slightly less likely to have completed secondary education compared to women in the control group (19.1% vs. 23.9%) (*p* = 0.07). All other demographic variables, including marital status, partner’s age, employment status, number of children and assets were balanced between the arms.

**Table 3:** Baseline balance between intervention and control group on key socio-demographic variables

Socio-demographic variable	Total (*n* = 1061)		Intervention (*n* = 533)		Control (*n* = 528)		Difference^##^
	n	% or mean (SD)	n	% or mean (SD)	n	% or mean (SD)	
**Woman’s age**	1061	33.0 (6.4)	533	33.4 (6.3)	528	32.7 (6.5)	0.09*
**Partner’s age**	1061	39.7 (9.18)	531	40.0 (8.8)	521	39.4 (9.5)	0.23
**Education**							0.07*
No education	408	38.5%	221	41.6%	187	35.5%	
Some primary (complete/incomplete)	424	40.0%	210	39.3%	214	40.6%	
Secondary	228	21.5%	102	19.1%	126	23.9%	
**Marital status**							0.84
Married	491	46.3%	242	45.6%	249	47.3%	
Partnered, live together	541	50.1%	276	52.0%	265	50.4%	
Partnered, do not live together	25	2.4%	13	2.5%	12	2.3%	
**Partner employment status**							0.30
Income-generating activities (self-employed, formally employed or other)	626	59.3%	323	60.8%	303	57.7%	
Does not work	430	40.7%	208	39.2%	222	42.3%	
**Number of people in household**	1061	6.8(6.6)	533	6.8 (2.3)	528	6.7 (2.3)	0.38
**Number of children in household**	1061	4.2(1.8)	533	4.3(1.9)	528	4.1(1.8)	0.14
**Childcare task sharing**							0.80
Partner shares childcare tasks	477	46.40%	241	46.8%	236	46.0%	
Woman or others alone	551	53.6%	274	53.2%	277	54.0%	
**Number of assets** ^**#**^	1061	5.5 (1.0)	533	5.4(1.1)	528	5.5(1.0)	0.64
**Religion**							0.23
Adventist	820	77.3%	422	79.2%	398	75.4%	
Protestant	143	13.5%	66	12.4%	77	14.6%	
Other	98	9.3%	45	8.4%	53	10.0%	

^#^12-item asset score created, based on household ownership of the following: mosquito net, radio, bicycle, agricultural land, mobile phone, iron, sewing machine, portable light, livestock (any), cookstove, plates, bed/mattress

^##^Chi-squared or t-test employed to test for significant differences between groups, with significance reported with

** if p </=0.05 or * if p </=0.10

Most outcomes at baseline (Table 4) were balanced; however, there was a significant difference in reports of emotional IPV, with the control experiencing significantly less (38.2% vs. 29.9%) (*p* = 0.005) than the intervention group at baseline. Both groups reported some IPV in the last six months, with emotional IPV reported at the highest rates, followed by physical or sexual IPV and then reproductive coercion. Both groups reported instances of NPV, with harassment more common than physical or sexual NPV and had similar levels of IPV, NPV, cookstove uptake, income generating activities, mental health scores, food insecurity, self-efficacy scores, and Ryff social agency scores at baseline.

**Table 4:** Key outcomes at baseline by intervention allocation

	Total (*n* = 1061)	Intervention (*n* = 533)		Control (*n* = 528)		Difference**
	% or mean (SD)	*n*	% or mean (SD)	*n*	% or mean (SD)	
**IPV past six months**						
Physical or sexual IPV	21.8%	124	23.3%	106	20.15%	0.21
Emotional IPV	34.1%	203	38.2%	157	29.9%	0.005**
Reproductive coercion	9.0%	48	9.3%	45	8.7%	0.77
**Non-partner violence past six months**						
Harassment	22.0%	120	22.5%	113	21.4%	0.66
Physical or sexual NPV	8.6%	51	9.6%	40	7.6%	0.25
**Uptake and use of clean cooking system**	22.1%	118	22.1%	117	22.1%	0.99
**Income generating activity**	19.6%	106	19.9%	102	19.3%	0.81
**Engagement in learning a skill**	Not assessed at baseline					
**Mental health**						
Depression/anxiety	5.4%	28	5.2%	29	5.5%	0.86
PTSD	5.2%	27	5.1%	28	5.3%	0.86
**Food insecurity** ^**#**^	10.9 (3.6)	533	10.8 (3.6)	528	11.1 (3.5)	0.24
**Self-efficacy score**	24.5 (4.12)	498	24.5(4.22)	484	24.5 (4.02)	0.80
**Shift and Persist mindset score**	Not assessed at baseline					
**Ryff social agency score**	18.40 (2.52)	476	18.36 (2.03)	478	18.44 (1.84)	0.56

Chi-squared or t-test employed to test for significant differences between groups

** if p </=0.05 or * if p </=0.10

^#^ Baseline rates were assessed using only 5/8 items of the HFIAS

At six months post intervention, 81.72% of the study participants were located and surveyed by the research team before expiry of the RNEC research permit deadline of August 2019. Table 5 presents primary and secondary outcomes at six months post-intervention. No significant differences were noted in incidents of IPV in the past six months in the intervention vs. the control group. For NPV, however, there appears to be a trend toward reduced experience of physical or sexual NPV at six months post intervention, with 5.7% of women in the intervention arm reporting experiencing NPV in the past six months compared to 8.18% in the control arm (aIRR: 0.65, (0.39–1.07); *p* = 0.091). In the assessment of mental health, we found significantly greater incidents of probable anxiety and/or depression (aIRR = 1.79 (1.00-3.22); *p* = 0.05) and probable PTSD (aIRR: 2.07 (1.10–3.91); *p* = 0.024) amongst women in the intervention group compared to the control group. The HSCL score was tested with a cut-off of 1.75 as suggested by Bass et al. [36] and found results remained significant at the 10% level.

**Table 5:** Primary and secondary outcomes

	Intervention Arm (*n* = 439)		Control Arm (*n* = 428)		Unadjusted		Adjusted^#^	
	Number of participants	Mean (SD) or percentage	Number of participants	Mean (SD) or percentage	IRR/ coefficient (95% CI)	*P* value	aIRR/ coefficient (95% CI)	*P* value
**IPV past six months**								
Physical or sexual IPV	53/409	12.96%	54/391	13.81%	0.94 (0.66–1.33)	0.724	0.80 (0.58–1.09)	0.161
Emotional IPV	95/408	23.3%	85/391	21.7%	1.17 (0.88–1.56)	0.288	0.95 (0.73–1.23)	0.688
Reproductive coercion	38/405	9.4%	25/389	6.4%	1.45 (0.90–2.37)	0.127	1.26 (0.79–2.01)	0.332
**NPV past six Months**								
Harassment	47/439	10.71%	42/428	9.81%	1.09 (0.73–1.62)	0.665	1.09 (0.74–1.60)	0.668
Physical or sexual NPV	25/439	5.7%	35/428	8.18%	0.70 (0.42–1.14)	0.153	0.65 (0.39–1.07)	0.091*
**Verified uptake and use of clean cooking systems**	282/445	63.37%	289/448	64.51%	0.95 (0.88–1.02)	0.176	0.95 (0.88–1.01)	0.113
**Income- generating activity in the past six months**	145/439	33.03%	116/428	27.10%	1.22 (0.99–1.50)	0.05*	1.25 (1.04–1.50)	0.018**
**Engagement in skill learning**	268/407	65.85%	171/404	43.55%	1.56 (1.36–1.77)	0.000**	1.59 (1.39–1.82)	0.000**
**Mental health**								
Depression/Anxiety	29/439	6.61%	16/428	3.74%	1.77 (0.97–3.21)	0.061*	1.79 (1.00-3.22)	0.050**
PTSD	27/439	6.15%	13/428	3.04%	2.02 (1.06–3.87)	0.033**	2.07 (1.10–3.91)	0.024**
**Food insecurity** ^**^**^	439	12.69 (5.50)	428	12.81 (5.41)	0.99 (0.93–1.05)	0.707	0.98 (0.93–1.03)	0.396
**Self-efficacy score**	425	25.22 (3.90)	404	24.82 (3.90)	1.02 (0.99–1.04)	0.143	1.02 (1.00-1.04)	0.103*
**Shift and Persist score**	439	27.67 (3.85)	429	27.20 (4.29)	1.02 (1.00-1.04)	0.092*	1.02 (1.00-1.04)	0.040**
**Ryff social agency**	398	19.02 (2.10)	390	19.12 (2.09)	0.99 (0.98–1.01)	0.480	1.00 (0.98–1.02)	0.901

** if p </=0.05 or * if p </=0.10

^#^All outcomes adjusted for baseline value of the outcome (except Shift and Persist and skill learning which were not assessed at baseline), woman’s age at baseline, woman’s education, and experience of emotional IPV at baseline

^ Results are similar when a binary indicator of severe food insecurity was created. (Coates et al. 2007)

Significant improvements were noted in self-reported engagement in income generating activities (aIRR = 1.25 (1.04–1.50); *p* = 0.018) and engagement in skill building (aIRR = 1.56 (1.36–1.77); *p* < 0.001). There were significant differences in measures of self-efficacy and the ability to manage stressful situations (Shift and Persist scale); however, the effect sizes were very small. No significant differences were seen between women in the intervention and control arm in their measures of social agency, food insecurity, experience of harassment, reproductive coercion, or uptake of clean cooking systems.

**Table 6:** Ancillary (conditional) analysis

Outcome	*N*	IRR/adjusted coefficient (95% CI)	*P* value
Physical or sexual IPV amongst those who experienced any IPV at baseline ^#^	*n* = 402	0.71 (0.49 to 1.01)	0.056*
Past month income amongst those working in the past 6 months^#^	*n* = 254	1.34 (0.99 to 1.82)	0.059*
Use of woman’s physical punishment towards children amongst those with children^#, $^	*n* = 844	1.06 (1.00 to 1.13)	0.054*
Women’s report of men’s participation in childcare^#, $^	*n* = 775	1.29 (1.05 to 1.58)	0.017**

** if p </=0.05 or * if p </=0.10

^#^Adjusting for baseline values of emotional violence, woman’s education, woman’s age

^$^Results hold after adjusting for baseline value of childcare task sharing

Table 6 reports outcomes beyond our primary analysis plan. The four ancillary analyses included physical and sexual IPV amongst those who experienced IPV at baseline, income in the past month for those working, women’s use of physical punishment towards children (amongst those with children), and women’s report of partner’s participation in childcare. These were exploratory in nature and reflect the change in literature that occurred between the initiation of the study and its endline analysis.

The ancillary analyses of individuals who had reported experience of IPV at baseline suggests a significant reduction in physical or sexual IPV because of the personal agency training, but no effects on preventing IPV amongst those who were not already experiencing IPV at baseline.

Past-month income amongst those working improved with the personal agency training. While use of physical disciplinary tactics and men’s participation in childcare was not initially planned for, this was also added as an exploratory outcome as this was assessed in a recent study by Doyle et al. (2018) [12]. At follow-up, 82% of women reported using at least one form of physical punishment against their child and overall, we find that the intervention arm reported a slightly greater use of physical punishment towards children. At the same time, we find that women in the intervention arm are more likely to report that their partner participated in childcare equally or took this responsibility most of the time.

### Study limitations

This study faced several limitations due to being conducted in a humanitarian setting. Our research activities were often constrained due to security issues affecting entry of research staff into the camp and our contacts were limited to the field team at Plan International that had access to the camp. There were significant policy changes during this study including a ban on all firewood distribution and the institutionalization of a cash for fuel program. These changes can potentially mitigate our ability to measure the impacts of the intervention by changing the prevalence of outcomes such as cookstove uptake and IPV. The national regulatory authority overseeing all camp research and program activities moved from MIDIMAR to MINEMA, requiring a re-approval process for the study. Participants were able to move freely outside of the camp at a greater rate than originally anticipated, resulting in a larger loss to follow up than expected. Randomization was done at the individual level and due to the dense living arrangements for families within the camp, there is a risk of contamination between the study arms. Moreover, as the NMN trainers are residents of the camp, it is likely that non-participants may have learned about the training after the training deployment had been completed, that could result in an underestimate of effects. Moreover, many individuals had moved to other households due to marriage or change in their partnership status at follow-up leading to a large loss to follow-up.

Furthermore, some limitations were due to the limited funding for this study. The data was collected only six months after the intervention, restricting the conclusions regarding the longer-term impacts of the intervention on this population. Additionally, while the formative work and dialogue recognized that agency enhancement that excludes men may pose challenges for the women the program is intended to benefit, due to the small sample size, we were unable to cross-randomize and investigate the impact of the partner engagement component of the intervention.

## Conclusions

With more than 80 million people forcibly displaced worldwide due to conflict or other forms of persecution [33], it is important that interventions targeted to enhance women’s empowerment consider the extent of the issue and the limited resources available to achieve this aim. By abbreviating and adapting the SEE Change agency-enhancing intervention with a gender dialogue component that addresses socio-cultural norms and harmful stereotypes, this study aimed to move us closer to building the evidence-base for an integrated approach to addressing key economic and social well-being concerns for women in refugee settings. This is the first large-scale evaluation of a personal agency training that includes a male engagement component conducted within a post-conflict setting.

Our approach makes three key contributions. The first is to fill the gap on impacts of an abbreviated agency-based interventions on economic and overall well-being of women in humanitarian settings. The focused deployment (two days for women, one day for male partners) contrasts to the IMAGE intervention [19] implemented in phases over 12–15 months or Save the Children’s program *‘*Adolescents: Protagonists of Development’ [22] which included 60 + hours of empowerment and health content, 70 h of business-related content, deployed over several months. The second contribution was to establish that an abbreviated version of a personal agency training demonstrated significant improvements to livelihoods, despite no additional business content or cash transfer component. And the third was to measure NPV and integrate components that address it, such as increasing women’s agency and increasing clean cooking uptake, which can reduce women’s risk of experiencing opportunistic violence from non-partners during firewood/fuel collection.

We find significant impacts on uptake of income generating activities and skill building despite no focused content on business tools or development, similar to what has been seen in previous studies examining the longer personal agency training [24, 47]. Like Gibbs et al. (2020) [20], our exploratory analysis finds positive impacts on income generation, in line with increased income generating activities and skill building. However, little change was seen in self-efficacy or the Shift and Persist scores. Although significant, the percentage change in the Shift and Persist score was only 2%. Measures of social agency also did not change, in contrast to previous research showing positive impacts on psychometric measures. This lack of results on the pathway could be due to the abbreviated nature of the intervention or may be driven by the fact that these measures were not designed for this setting and lacked reliability and/or validity in this context.

Despite the economic outcomes, we found no overall significant impacts of the NMN intervention on experience of IPV in the last six months in the full sample. Descriptive statistics show that the overall rates of IPV reduced substantially during the study period, from 38 to 23%, as did rates of prevalence of depression and/or anxiety and PTSD. This is likely due to a simultaneous shift in cash-for-fuel policy deployed during the study period; previous research has shown that cash transfers can reduce rates of violence [48]. While our study was initially powered to detect a 35% reduction, this reduction in prevalence could be responsible for our study being underpowered to detect a reduction in IPV. These mixed results could be due to the overall reduction in GBV within the camp during the time that the study, or that the abbreviated nature of the intervention wasn’t sufficient to create the necessary change in behaviors with the study sample. However, the exploratory analyses demonstrate a significant reduction in experience of IPV on those who reported IPV at baseline. This finding is in line with findings by Dunkle et al. (2020) [13], who showed that at 24-months post follow-up, a couple intervention impacted IPV only amongst those who reported experiencing IPV at baseline. Similarly, Angelucci et al. (2022) [21] find impacts of their cash plus empowerment intervention on IPV only amongst those at high risk for IPV at baseline. Therefore, while the abbreviated intervention may not prevent IPV, it appears to reduce rates in those already experiencing it. These findings have implications for who should be targeted and who may be at increased risk for backlash from empowerment interventions.

The potential lack of effect of the empowerment intervention on cookstove uptake, while disappointing, is not surprising. The results could be driven by the possibility that due to our intervention women were potentially using the fuel cash transfer towards business generation. Alternatively, the intervention may have been too mild to impact uptake in the remaining 36% who were not using clean cooking solutions despite the cash for fuel policy. Increasing uptake of clean cookstoves is a complex matter, and within a humanitarian setting, even more so. Competing efforts from the multiple stakeholders (including UNHCR, NGOs, and MINEMA) supporting the camp gave rise to inconsistent and incomplete distribution of goods and services, making uptake of any one opportunity, such as clean cookstoves and fuels, more complicated.

Despite the lack of impact on clean cookstove uptake, NPV did seem to decrease in the intervention arm. As the effects did not come from increased cookstove uptake pathway, like Gulesci et al., (2021) [22] suggest, we can only hypothesize that these effects could come from a myriad of sources such as reduced exposure perhaps due to increased task sharing with their intimate partner (NMN resulted in greater engagement in childcare), greater social networks that help protect women from non-partner abuse, or through learning soft-skills such as better decision-making and planning that allows them to avoid potentially dangerous situations or being more assertive and self-confident when dealing with potential abusers.

Along with some positive findings, we also captured some unintended consequences such as a potential increase in use of harsh disciplinary approaches towards children and worsened mental health. Unlike Doyle et al. (2018) [12], who find a couples intervention with an emphasis on positive parenting resulted in reduced physical punishment towards children, our study which did not focus on parenting finds a slight increase. The percentage of women reporting use of any form of physical discipline against their child was significantly greater in the intervention arm compared to the control arm. In this population, use of force was common for the majority of respondents interviewed. The slight increase (6%) may be a function of increased stress due to women’s time spent on income generating activities. As this was not measured at baseline, we are unable to explore a change in score, nor can we confirm that at baseline there was no imbalance on this outcome. Given the overall high prevalence of such disciplinary tactics in this setting, we would like to highlight this as an area of future research.

Our findings also support previous literature where Green et al. (2015) hypothesize that despite extensive economic gains, their intervention too failed to improve mental health in conflict affected Uganda, due to the stress induced by generating business activities [32]. Our findings also reveal that for refugee populations that have experienced significant trauma, personal agency training may exacerbate mental health symptoms compared to the control group, for whom the prevalence of probable PTSD and depression and/or anxiety appear to have reduced over time. This outcome is rarely measured in studies that evaluate socio-emotional skills training with the aim of increasing income generation and is particularly important to measure amongst conflict-affected populations. Greater personal agency and motivation are likely resulting in greater introspection and a desire to achieve goals. This may potentially exacerbate symptoms of anxiety. We note that our sample consists of refugees in a protracted situation who have had time to settle into the camp and have also had access to mental health services which probably resulted in the low prevalence of probable PTSD [36], depression and/or anxiety that we see in our sample. While we do not know the history of mental health interventions received by our sample, we do know that the level of trauma experienced by the women in this population in the past is high. The act of psychological reflection and activities, such as the ‘Letting Go’ exercise, can trigger revisiting this trauma. Given that this training necessitates substantial self-reflection, we consider it appropriate for implementation in protracted refugee settings. However, we advise exercising caution when introducing these concepts in acute humanitarian settings. Still, these results provide important evidence that personal agency interventions deployed in conflict-affected populations must be adapted to include more trauma-informed exercises and be accompanied by sufficient psychological support systems.

Overall, we recommend integrating personal agency interventions, along with socio-emotional and business empowerment interventions, with psychosocial support and evidence-based mental health interventions for refugee women. With refugee populations, the evidence-base for shorter, transdiagnostic, group-based, indicated mental health prevention programs that are implemented by non-specialists is emerging. For example, recent evidence supports the effectiveness of Self-Help Plus, a five-session acceptance and commitment therapy-based intervention with refugees in Uganda [49]. The intervention promotes psychological flexibility and helps people identify and behave in line with their values, which has similarities to the approaches used in NMN to enhance personal agency. A recent review emphasizes the necessity for interventions to be firmly rooted in the local context that facilitate exploration of the complexity of each woman’s situation to address her multifaceted needs across various life domains [50].

The lack of overall reduction in IPV may be due to the short duration of the intervention. It may also be due to the fact that agency training may reduce existing cases of IPV but cannot prevent IPV amongst those who were not experiencing it at the time of receiving the training. It is also possible that for women experiencing an increase in PTSD symptoms, particularly those related to re-experiencing, this intervention may increase their perpetration of psychological IPV towards their partner and hence increase women’s own risk of IPV revictimization [51] resulting in an average null effect of the intervention on IPV. These findings, however, strongly suggest that trauma-affected populations continue to be at increased risk of mental illness, and any intervention with these populations must assess and address mental health. This study highlights the need for innovative behavioral interventions designed for low-resource settings that promote livelihoods and address social challenges. It is essential to assess potential negative outcomes within personal agency interventions, to monitor and address any issues that may arise during the program. In addition, it would be useful to consider extending the intervention, either by expanding its content or supplementing the program with follow up sessions. Future research should focus on developing effective interventions that integrate mental health and psychosocial support to promote long-term empowerment and reduce the risk of IPV in refugee populations.

## Data Availability

Due to the sensitive nature of the data, the dataset used and/or analyzed during the current study can be made available from the corresponding author on reasonable request and after IRB approval has been obtained.
